# Predicting the prevalence of type 2 diabetes in Brazil: a modeling study

**DOI:** 10.3389/fpubh.2024.1275167

**Published:** 2024-05-02

**Authors:** Patrícia Vasconcelos Leitão Moreira, Adélia da Costa Pereira de Arruda Neta, Flávia Emília Leite Lima Ferreira, Jevuks Matheus de Araújo, Rômulo Eufrosino de Alencar Rodrigues, Rafaela Lira Formiga Cavalcanti de Lima, Rodrigo Pinheiro de Toledo Vianna, José Moreira da Silva Neto, Martin O’Flaherty

**Affiliations:** ^1^Department of Nutrition, Federal University of Paraiba, João Pessoa, Paraíba, Brazil; ^2^Department of Economy, Federal University of Paraiba, João Pessoa, Paraíba, Brazil; ^3^Technical School of Health of the Federal University of Paraíba, João Pessoa, Paraíba, Brazil; ^4^Department of Public Health and Policy, University of Liverpool, Liverpool, United Kingdom

**Keywords:** type 2 diabetes prevalence, demographic changes, obesity trends, projection, target strategies, modeling

## Abstract

**Aims:**

We adopted a modeling approach to predict the likely future prevalence of type 2 diabetes, taking into account demographic changes and trends in obesity and smoking in Brazil. We then used the model to estimate the likely future impact of different policy scenarios, such as policies to reduce obesity.

**Methods:**

The IMPACT TYPE 2 DIABETES model uses a Markov approach to integrate population, obesity, and smoking trends to estimate future type 2 diabetes prevalence. We developed a model for the Brazilian population from 2006 to 2036. Data on the Brazilian population in relation to sex and age were collected from the Brazilian Institute of Geography and Statistics, and data on the prevalence of type 2 diabetes, obesity, and smoking were collected from the Surveillance of Risk and Protection Factors for Chronic Diseases by Telephone Survey (VIGITEL).

**Results:**

The observed prevalence of type 2 diabetes among Brazilians aged over 25 years was 10.8% (5.2–14.3%) in 2006, increasing to 13.7% (6.9–18.4%) in 2020. Between 2006 and 2020, the observed prevalence in men increased from 11.0 to 19.1% and women from 10.6 to 21.3%. The model forecasts a dramatic rise in prevalence by 2036 (27.0% overall, 17.1% in men and 35.9% in women). However, if obesity prevalence declines by 1% per year from 2020 to 2036 (Scenario 1), the prevalence of diabetes decreases from 26.3 to 23.7, which represents approximately a 10.0% drop in 16 years. If obesity declined by 5% per year in 16 years as an optimistic target (Scenario 2), the prevalence of diabetes decreased from 26.3 to 21.2, representing a 19.4% drop in diabetes prevalence.

**Conclusion:**

The model predicts an increase in the prevalence of type 2 diabetes in Brazil. Even with ambitious targets to reduce obesity prevalence, type 2 diabetes in Brazil will continue to have a large impact on Brazilian public health.

## 1 Introduction

Diabetes Mellitus (DM) is a chronic metabolic disease that leads, over time, to serious damage to the heart, blood vessels, eyes, kidneys, and nerves (1). Type 2 diabetes (T2D) is the most common, which usually affects adults (1). The prevalence of type 2 diabetes has increased dramatically over the past three decades in countries of all income levels (1). In Brazil, the estimated prevalence of type 2 diabetes is 9.2%, ranging from 6.3% in the North to 12.8% in the Southeast (2).

Risks of type 2 diabetes increase in obese individuals (3), with obesity being an independent risk factor for type 2 diabetes (4). According to the World Health Organization (WHO), the number of adults with obesity has increased more than seven times since 1975 (5). In Brazil, obesity prevalence in adults more than doubled between 2003 and 2019, reaching 26.8%. In the same period, obesity also doubles its prevalence, with men and women prevalence at 30.2 and 22.8%, respectively (6).

In addition, there is a positive association between smoking and the incidence of diabetes (7, 8), with smokers being 30 to 40% more likely to develop type 2 diabetes than those who do not smoke (9, 10). In Brazil, the total percentage of smokers aged 18 years or over is 9.5%, with 11.7% among men and 7.6% among women. Globally, smoking alone is one of the leading causes of preventable disease and death (11).

Obesity and diabetes substantially impact healthcare costs, with 30% of the Unified Health System (*Sistema Único de Saúde – SUS*) cost attributable to diabetes and 11% attributable to obesity (12). Research showed that the total cost to the health system attributable to smoking is 23.3 billion reais per year (13).

Estimates of current and future type 2 diabetes prevalence are essential for managing *SUS* resources and encouraging intensive intervention measures in relation to risk factors to counteract trends of increasing prevalence (14, 15). In the context of Brazil, such projections are characterized by their scarcity and lack of precision since most estimates, such as those produced by the Diabetes Atlas of the International Diabetes Federation, are based on urbanization trends and demographic changes only, without taking into account trends in diabetes risk factors. In this way, forecasts based on trends in key risk factors appear to be more realistic.

The aim of the study was to use the type 2 diabetes-IMPACT model (14) with data from VIGITEL BRASIL and to describe trends in the type 2 diabetes-IMPACT (14) model with data from VIGITEL BRASIL and describe trends in type 2 diabetes and key risk factors, using the model to predict the likely future prevalence of type 2 diabetes, taking into account demographic changes and trends in key risk factors: obesity and smoking. Finally, it uses the model to estimate the likely future impact of different policy scenarios, such as policies to reduce obesity.

## 2 Materials and methods

### 2.1 Model overview

The model is a multistate Markov model that brings together information on population trends, obesity, and smoking at a given time to estimate the prevalence of diabetes in the future. It was previously used to estimate future diabetes prevalence in Tunisia, Turkey, Palestine, and Saudi Arabia (14, 16–19).

**Figure 1:**
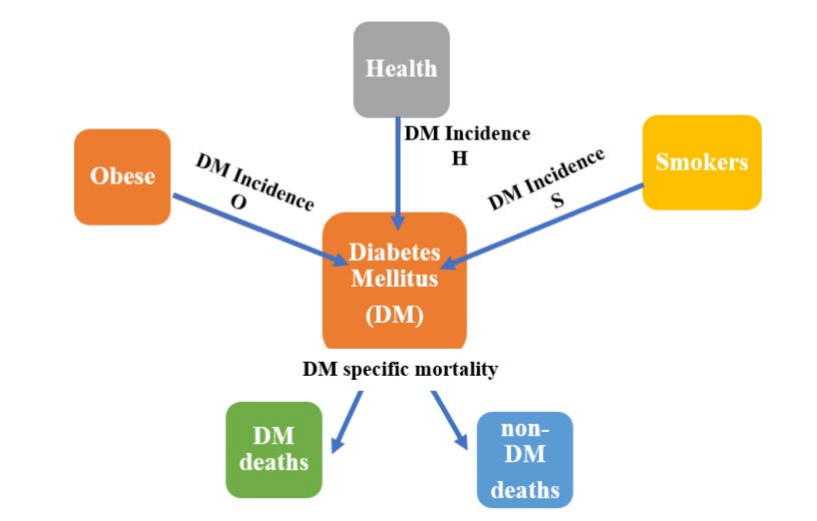
The type 2 diabetes model structure.

The total population is divided into several pools: type 2 diabetes, obese, smokers, and “healthy” (i.e., non-obese, non-smoking, non-diabetic) (Figure 1). Population demographic trends are used to inform the relative size of the “starting states,” and transition probabilities are used to estimate the proportion of persons moving from the starting states to the type 2 diabetes and death states. There are two “absorbing states”: type 2 diabetes-related death and non-type 2 diabetes-related deaths as competing risks for mortality. Potential overlaps between the healthy, obese, and smoking groups are estimated by calculating the conditional probabilities of membership, which allows for estimating what proportion of diabetic new cases can be attributed to smoking and obesity at each cycle.

### 2.2 Data sources

Data on the Brazilian population in relation to sex and age were collected from the Brazilian Institute of Geography and Statistics (*Instituto Brasileiro de Geografia e Estatística – IBGE*), data from the 2010/2060 population projection, published in 2018 (20).

Data on the prevalence of type 2 diabetes, obesity, and smoking were collected from the Surveillance of Risk and Protection Factors for Chronic Diseases by Telephone Survey (VIGITEL) (21), studies that make up the Surveillance of Risk Factors for Chronic Non-communicable Diseases (NCDs) of the Ministry of Health. Cases of type 2 diabetes are self-reported by individuals surveyed who already have a previous diagnosis of diabetes. The prevalence of obesity is obtained from self-reported data on weight and height to calculate the Body Mass Index (BMI), and individuals with a BMI >30 kg/m^2^ are considered obese. Individuals who use cigarettes have their smoking status registered.

### 2.3 Estimating the incidence, case fatality, and mortality parameters

We estimated type 2 diabetes incidence for the Brazilian population in 2010 using DISMOD, a freely available software (22). To estimate diabetes incidence, case fatality rates, and mortality rates, we used the following as inputs for DISMOD diabetes prevalence in 2010: diabetes mellitus remission rate and diabetes mellitus relative risk for mortality.

We assumed that the diabetes mellitus remission rate is zero, and the relative risk for mortality can be estimated with the method proposed by Barendregt et al. (23), based on the usual RR for mortality and disease prevalence, using this formula:


$$\text{RRadj}=\frac{RR}{p.RR+1-p}$$

Where *RRadj* is the relative risk of mortality, *RR* is the usual relative risk for mortality (mortality diseased/mortality healthy) (24), and *p* is disease prevalence.

The potential overlaps between the model health states were handled in three different ways. First, smoking prevalence was multiplied by obesity prevalence to estimate the proportion of the population who were both obese and smokers. Then, such a proportion was subtracted from the ‘original’ smoking prevalence to leave in the (Smokers) state only those individuals who were smokers but not obese. Second, we estimated the number of individuals with T2DM (in the Diabetes state) in whom the disease was assumed to be ‘caused’ by obesity as exposure by multiplying the ‘*population attributable risk*’ by the size of (Diabetes) state (25). Then, the number of such individuals was subtracted from the total obese individuals in the population to leave in the (Obese) state only those obese individuals who do not have T2DM. Finally, we applied the same previous approach of the *population attributable risk* to leave in the (Smokers) states only those people who are smokers but do not have T2DM.

### 2.4 Validation

Model validation is very important in any modeling exercise. The model developed for Brazil involves the years 2006 to 2036, with the base prevalence in 2006. As we have data available on the prevalence of diabetes in the country from 2006 to 2020, we compared the model results with the prevalence estimates observed for each year of type 2 diabetes cases at VIGITEL. Type 2 diabetes is diagnosed as self-reported by the population within the study with a previous diagnosis. Thus, we set a correction factor of 1.5, as these cases may underestimate the actual prevalence of the disease since Muzy et al. (2) show that the proportion of underreported cases reaches 50% in Brazil. We then found an equivalence between the model data output and the estimated prevalence demonstrated in the VIGITEL data (validation results are in the Technical Appendix).

### 2.5 Type 2 diabetes forecast and policy scenarios

Effective policy decisions can be modeled by the estimated intervention effect on trends in risk factors, and the trend parameter can be modified to model increasing, decreasing, or stable trends in the prevalence of obesity and/or smoking.

First, we present predictions for type 2 diabetes prevalence and burden through 2036 (30 years from our initial prevalence in 2006), assuming current trends in obesity and smoking continue. Obesity and smoking trends were conducted from the population data divided by age groups, and the data were grouped into six age groups. The projection used a pooled OLS model to control for the fixed-time effect. The pooled data allows you to increase the amount of information and control the effect of time. Next, we explored the potential effect of population-level interventions to reduce obesity.

### 2.6 Policy scenarios

First, we present forecasts for type 2 diabetes prevalence and the burden up to 2036 (26 years from our initial prevalence estimate in 2010), if current trends in obesity and smoking continue as our baseline scenario.

Then, we explored the potential effect of population-level changes in obesity in our first intervention; we are proposing a 1% reduction per year in the prevalence of obesity from the year 2020 to the year 2036, verifying the impact on the diabetes burden. In our second intervention, in a more optimistic scenario, we propose a 5% reduction per year in the prevalence of obesity from the year 2020 to the year 2036, verifying the impact on the diabetes burden.

### 2.7 Sensitivity analysis

The analysis of the extreme method (Briggs) was used (26), which consists of running the model with all parameters adjusted to realistic minimum and maximum values, carried out in Excel; and consists of a very conservative approach, but which allows for a more transparent understanding of the weight of each parameter concerning the model results.

## 3 Results

### 3.1 Demographic and epidemiological chances

Brazil has approximately 213 million inhabitants. We considered the population aged over 25 years. For this age group, the number of men is estimated to grow from 64 million in 2020 to 76 million in 2036 and from 70 to 84 million for women. The proportion of young people aged 25–34 years will decrease from 25.4% in 2020 to 18.6% in 2036 in relation to the total population of the country. By 2036, 22.8% of the population 25 years and above will be aged over 65 years, compared with 15.3% in 2020 (Table 1).

**Table 1:** Projection data of the Brazilian population from 2006 to 2036.

Age groups	2006			2020			2036		
	Population N^*^ (million)			Population N^*^ (million)			Population N^*^ (million)		
	M**	W***	%****	M**	W***	%****	M**	W***	%****
25–34	15.6	15.5	31.0	17.6	17.1	25.4	15.0	14.6	18.6
35–44	12.7	13.0	25.6	15.9	16.7	24.5	16.6	16.7	20.8
45–54	9.6	10.1	19.6	12.6	13.6	19.5	16.0	16.9	20.6
55–64	5.8	6.5	12.2	9.6	10.9	15.3	13.0	14.5	17.2
65–74	3.2	3.9	7.2	5.7	7.0	9.4	9.2	11.1	12.7
75+	1.7	2.6	4.3	3.2	4.8	5.9	6.4	9.7	10.1
Total	48.8	51.9	100.0	64.1	70.3	100.0	76.4	83.7	100.0

N, number; M, men; W, women; %: percentage in relation to the total population.

### 3.2 Observed prevalence of type 2 diabetes, obesity, and smoking

The observed prevalence of type 2 diabetes in Brazil in 2006 among the Brazilian population aged 25 years and above was overall 10.8% (min./max: 5.2–14.3); 11.0% (min./max:2.2–17.4) in men and 10.6% (min./max: 1.7–17.8) in women. The observed prevalence of type 2 diabetes in Brazil in 2020 was overall 13.7% (min./max: 6.9–18.4); 19.1% (min./max: 2.4–23.0) in men and 21.3% (min./max: 2.9–22.2) in women. Obesity is a common risk factor, with 14.5% prevalence among men and 12.5% among women in 2006. This prevalence increased to 22.9% among men and 21.7% among women, respectively, in 2020. Smoking prevalence was decreasing, from 17.8% (2006) to 12.8% (2020) among men and from 11.4% (2006) to 7.9% among women (Table 2).

**Table 2:** Brazilian population aged 25 years and above, obesity, and smoking prevalence by sex and age groups in 2006 and 2020.

Age groups	2006						2020					
	T2D %		Obesity %		Smoking %		T2D %		Obesity %		Smoking %	
	M	W	M	W	M	W	M	W	M	W	M	W
25–34	1.3	1.0	12.2	7.1	16.7	8.4	1.7	2.2	20.1	18.0	12.8	5.5
35–44	2.6	2.4	15.5	10.5	17.5	12.4	3.4	3.8	25.9	22.4	11.4	7.4
45–54	7.0	5.9	17.3	14.8	22.6	17.6	8.0	7.8	25.5	23.1	15.2	11.4
55–64	12.6	13.5	16.1	19.0	18.5	11.3	15.8	16.0	24.4	23.5	15.4	10.6
65–74	16.9	18.4	13.2	19.0	13.8	7.8	22.6	22.8	19.7	23.7	11.2	6.4
75+	18.3	17.7	8.9	18.3	8.3	5.4	24.6	22.7	14.2	20.8	6.5	4.4
Total	11.0	10.6	14.5	12.5	17.8	11.4	19.1	21.3	22.9	21.7	12.8	7.9

N, number; M, men; W, women.

### 3.3 The effect of obesity and smoking trends on type 2 diabetes prevalence

Changes in the prevalence of obesity and smoking were assumed to be linear, with varying degrees among men and women and in different age groups.

Obesity prevalence rose from 22.9% in 2020 to 31.1% in 2036 in men (annual increase of 0.51%) and from 21.7 to 29.1% in women (annual increase of 0.46%). Obesity prevalence was higher among men than women, with the highest prevalence observed among men aged 35-44, 45-54, and 55-64, and among women aged 55-64 and 65-74. The smoking trends showed a decrease in men and women. The projected prevalence for 2036 is 3.3% in men and 1.8% in women, representing a decrease of almost 82% in men and 84% in women. In some age groups, both for men and women, there is a tendency for the prevalence to be almost non-existent.

Assuming these annual trends in risk factors continue, the forecast prevalence of type 2 diabetes for 2036 is overall 27.0% (min 26.9–max 27.7): 17.1% in men (min 15.8–max 17.7) and 35.9% in women (min 31.1–max 37.7). The total number of Brazilian people with type 2 diabetes is projected to rise from 9 million in 2006 to 43 million in 2036, an increase of almost 400%. Diabetes prevalence is predicted to increase rapidly between 2006 (8.9%) and 2020 (26.3%), and then the increase starts to slow down in men. This trend, however, was not observed for women, who showed an ever-increasing trend in the prevalence of type 2 diabetes.

### 3.4 Scenario projections

If trends in obesity start to decline by 1% per year from 2020 to 2036 (Scenario 1), the prevalence of diabetes decreases from 26.3 to 23.7, representing approximately a 10.0% drop in 16 years. This would prevent approximately 5.2 million Brazilians from developing type 2 diabetes, 4.7 million of whom are women and 0.5 million men. If obesity declined by 5% per year in 16 years as an optimistic target (Scenario 2), the prevalence of diabetes decreased from 26.3 to 21.2, representing a 19.4% drop in diabetes prevalence. It is noteworthy that if only women were observed in the disease projection scenario for 2036, between baseline (35.9%) and Scenario 2 (25.8%), there could be a reduction of more than 30% in the prevalence of diabetes in this group, if obesity were reduced (Table 3). This corresponds to approximately 9.3 million Brazilians who would stop developing type 2 diabetes, 0.9 million men and 8.5 million women. Detailed results with sensitivity analysis are shown in Table 3.

**Table 3:** Scenario projection rate of prevalence of type 2 diabetes by gender with sensitivity analysis (Minimum-Maximum).

Baseline	Men %	Women %	Total %
2006	8.7 (6.9–10.4)	9.1 (7.3–10.9)	8.9 (7.1–10.7)
2020	20.4 (17.6–22.4)	31.7 (30.9–34.0)	26.3 (26.2–26.8)
2036	**17.1 (15.8–17.7)**	**35.9 (31.1–37.7)**	**27.0 (26.9–27.7)**
Scenario 1	Men %	Women %	Total %
2006	8.7 (6.9–10.4)	9.1 (7.3–10.9)	8.9 (7.1–10.7)
2020	20.4 (17.6–22.4)	31.7 (30.9–34.0)	26.3 (26.2–26.8)
2036	**16.4 (15.4–16.6)**	**30.2 (29.0–33.4)**	**23.7 (23.2–24.9)**
Scenario 2	Men %	Women %	Total %
2006	8.7 (6.9–10.4)	9.1 (7.3–10.9)	8.9 (7.1–10.7)
2020	20.4 (17.6–22.4)	31.7 (30.9–34.0)	26.3 (26.2–26.8)
2036	**15.9 (15.1–15.9)**	**25.8 (22.4–31.3)**	**21.2 (19.4–23.7)**

Projected prevalence of T2D by 2036.

## 4 Discussion

The forecast prevalence of type 2 diabetes for 2036 is 27.0% overall (17.1% in men and 35.9% in women), with almost 400% increase in the number of persons with type 2 diabetes between 2006 and 2036. If the two proposed scenarios for decreasing the prevalence of obesity were achieved, we would have a 10.0–19.4% (12.2 and 21.48%) reduction in the prevalence of type 2 diabetes by the year 2036. Because the smoking trend among Brazilians for the year 2036 is decreasing, and in some age groups, it will be practically nil, we chose not to include smoking within the proposed scenarios, as the effect would be very small on the prevalence of diabetes.

Obesity is a global epidemic, and rates are particularly high in the USA (42.4% in 2017/2018) (27) and Canada (57.1% in Ontario and 56.2% in Québec) (28) compared with 11.8–31.3% in India (29). In Brazil, the prevalence of obesity has also been growing over the years (30), which has many implications for chronic diseases, including type 2 diabetes. The factors associated with the increase in the prevalence of obesity are multidimensional, related both to the demographic transition and to social inequalities (31, 32) that reflect the high prevalence of food insecurity and the double burden of malnutrition of populations in developing countries, as is the case in Brazil (33, 34).

In developing countries such as Palestine, diabetes mellitus prevalence estimated by the model forecasts was 20.8% for 2020 and 23.4% for 2030 (16), while in Tunisia, the model forecasts a dramatic rise in prevalence by 2027 (26.6% overall, 28.6% in men and 24.7% in women) (14). In Qatar, type 2 diabetes prevalence increased from 16.7% in 2016 to 24.0% in 2050 in the baseline scenario (17). Studies show that if there is a control of the obesity epidemic in these countries, as well as a decrease in the prevalence of smoking, there is a probability of a decrease in the prevalence of DM2 (14, 16–19). In the present model, following the proposed scenarios for the reduction of obesity over the years, a decrease in the prevalence of type 2 diabetes will also be observed in Brazil.

### 4.1 Policy scenarios and their importance

This study proposes two intervention options and assesses their impact on future diabetes prevalence. In Brazil, obesity prevalence is increasing and is predicted to continue to increase. The frequency of overweight adults between 2006 and 2020 ranged from 42.6% in 2006 to 57.5% in 2020 (average increase of 1.04 pp./year). This increase was observed in both sexes, with the highest increase among women, ranging from 38.5% in 2006 to 56.2% in 2020 (1.24 pp./year) (35).

Brazilians have a national strategy for preventing non-communicable diseases. However, this strategy did not set a target for obesity reduction; instead, the strategy is limited to promoting a stabilization of obesity in the country by the year 2030 (36). With this desirable stabilization in mind, our model tends to be more daring, proposing a more plausible scenario of a 1% reduction per year in the prevalence of obesity and a more challenging scenario of a 5% reduction per year in the prevalence of obesity. Among the goals proposed to stop this growth of obesity and, consequently, of NCDs are the encouragement to practice physical activity in leisure time, increase the consumption of fruits and vegetables by 30%, and stop the consumption of ultra-processed foods (36).

Some initiatives for primary prevention of obesity have been implemented. The National Health Promotion Policy can be considered as a potential inducer of obesity prevention and control actions because it establishes as priority themes: (a) the development of actions for the Promotion of Adequate and Healthy Food (PAHF); (b) the promotion of food and nutrition security, aiming to contribute to the guarantee of the Human Right to Adequate Food; (c) encouragement of bodily practices and physical activity, providing public spaces to enable bodily activities that enhance comprehensive healthcare (37, 38).

Recently, at the national level, “Proteja” was launched, the National Strategy Plan for Prevention and Care of Child Obesity, with several essential strategies that must be implemented by Brazilian municipalities, such as monitoring the nutritional status and markers of children’s food consumption, institutional campaigns in the media mass communication on childhood obesity, and ensuring healthy canteens in schools, among other actions (39).

In the National Primary Care Policy (40), two programs with a potential impact on obesity stand out: the School Health Program (*Programa Saúde na Escola—PSE*) and the Health Academy Program (*Programa Academia da Saúde*), which encourages the practice of physical activity outdoors. Furthermore, Brazilian researchers have developed an exemplary proposal regarding healthier eating guidelines for their population, which is a reference for many countries around the world (41), just as there are intersectoral articulation initiatives for the prevention and control of obesity (42), so the scenarios proposed in this study are feasible and can be considered.

Regarding the program that encourages the practice of physical activity, in an evaluation of VIGITEL data in adults and the older adult, it was observed that increased frequency and level of leisure-time physical activity in adults are protective factors in relation to obesity (43). Another factor that can help to reduce the prevalence of obesity is the reduction of sedentary behavior. This has been strongly associated with reducing the occurrence of type 2 diabetes and cardiovascular disease. Last year, a physical activity guide for the Brazilian population was released (44).

Nguyen et al. (45), when building a model (ACE-Obesity Policy Model) simulating BMI, physical activity, consumption of fruits and vegetables, and incorporating the variable sedentary behavior in the Australian population, observed through the most realistic scenario that, if the population spent less than 4 h a day sitting, 3,204 deaths could be avoided per year, and among these 22% of deaths from type 2 diabetes, in addition to preventing the incidence of this disease by 58%. López-González et al. (46), evaluating the consumption of fruits and vegetables in a longitudinal study in adults, revealed that the increase of 100 g in the consumption of these foods in 1 year promoted a significant reduction in glycemic levels in the body weight and waist circumference. These studies provide a basis for nutritional education that should be carried out both within schools and in the practice of population self-care in primary healthcare.

### 4.2 Strengths and limitations

This is the first modeling study involving type 2 diabetes and risk factors for the Brazilian population. In addition, the projections that were carried out used data from an annual study that took place in the country uninterruptedly from 2006 to 2020, which brings greater security in projecting, in view of the trend of real data observed by long series.

A possible limitation of the study was the lack of inclusion of other possible risk factors that are related to diabetes were not considered, such as physical inactivity and poor diet. The use of studies that use self-reporting could also be questioned. However, in Brazil, given the geographic extent of the country, VIGITEL studies have been of high value for annual monitoring of the country’s epidemiological situation in relation to chronic non-communicable diseases and related risk factors. In this study, we sought to address this self-report issue in relation to the diagnosis of diabetes, using a correction factor of 1.5, which minimizes the harm of underestimation. Despite these limitations, the model used published prevalence data for obesity and smoking over a 15-year time series, which increases the likelihood of a more realistic trend estimate.

Small and possible variations in population estimates or the groups analyzed do not make the results unfeasible or biased.

### 4.3 Public health implications: a call to actions

Diabetes prevention can be achieved through measures that focus on reducing obesity. Currently, in the country, the discussion of some of these measures is gaining momentum. Some have not yet been implemented, but we can see successful examples in other countries, such as the taxation of junk food, including sweet beverages, clear nutrition labeling, with interest in front-of-pack (FOP) labeling policies based on comprehensive nutrient profiling models, public awareness, regulation of food advertising (especially targeted to children), and school-based health promotion initiatives (47–49). Similarly, the fight against social inequalities needs to be articulated with the obesity reduction action plan since it is proven that socioeconomic factors such as unemployment and poverty are associated with the increase in the prevalence of obesity (50, 51).

Recently, some modeling studies have observed the effect of taxation on foods, especially ultra-processed foods, and the benefit of reducing the prevalence of obesity and other diseases (52, 53). A study developed by Passos et al. (53) showed that a 1% increase in the prices of ultra-processed would lead to a decrease in the prevalence of overweight and obesity (0.33 and 0.59%, respectively), and this effect is even greater in low-income populations (0.34 and 0.63%).

Among the ultra-processed products, a group that deserves special attention is the sugar-sweetened beverages (SSBs), which include soft drinks and industrialized juices, as the relationship between the consumption of SSBs and type 2 diabetes is now supported by substantial epidemiological evidence (54, 55). High levels of free sugars in the diet increase the risk of obesity and diabetes (56, 57). Sugary drinks are often responsible for a large part of the free sugar consumed (58) and have been the main focus of policies (59). Then, a high SSB tax might be an effective fiscal policy to decrease the purchase and consumption of SSB and reduce overweight/obesity prevalence, especially if the tax were specific for beverage volume and in upper-middle- and middle-income countries, such as Brazil (60).

In a recent meta-analysis, economic tools, product reformulation, and environmental measures were effective in reducing sugar intake or weight outcomes, while labeling, education, and interventions combining educational and environmental measures found mixed effects. The most frequently implemented measures in Europe are public awareness, nutrition education, and labels (61). In addition to these strategies mentioned, Brazil has produced material to support health teams and professionals in the management of obesity in the Unified Health System, with emphasis on the collective approach (62).

## 5 Conclusion

Our study brings promising results for the reduction of type 2 diabetes with actions promoting the reduction of the prevalence of obesity in the Brazilian population in accordance with national policy and highlighting possible and innovative possibilities, such as the consideration of more optimistic scenarios. However, current initiatives will require substantial scaling up and adoption to have a significant impact on current obesity trends.

## Data availability statement

The original contributions presented in the study are included in the article/Supplementary material, further inquiries can be directed to the corresponding author.

## Ethics statement

The studies involving humans were approved by the Ethics Committee of Federal University of Paraíba. The studies were conducted in accordance with the local legislation and institutional requirements. The ethics committee/institutional review board waived the requirement of written informed consent for participation from the participants or the participants’ legal guardians/next of kin because these are secondary data made available on public domain sites of the Brazilian Unified Health System.

## Author contributions

PM: Conceptualization, Data curation, Formal analysis, Funding acquisition, Methodology, Project administration, Supervision, Validation, Writing – original draft, Writing – review & editing. AdA: Conceptualization, Data curation, Formal analysis, Methodology, Writing – original draft, Writing – review & editing. FF: Writing – original draft, Writing – review & editing. JdA: Formal analysis, Writing – original draft, Writing – review & editing. RR: Data curation, Formal analysis, Methodology, Writing – original draft. RL: Writing – original draft, Writing – review & editing. RV: Writing – original draft, Writing – review & editing. JS: Writing – original draft, Writing – review & editing. MO’F: Conceptualization, Formal analysis, Methodology, Supervision, Validation, Writing – original draft, Writing – review & editing.
