# Supplementary material for: Predicting the prevalence of type 2 diabetes in Brazil: a modeling study
Source: Front Public Health. 2024 May 2;12:1275167. doi: 10.3389/fpubh.2024.1275167 (PMC11096587; doi:10.3389/fpubh.2024.1275167)
Supplement: Supplementary file 1 [file Data_Sheet_1.PDF]

# TECHNICAL APPENDIX

## PREDICTING THE PREVALENCE OF TYPE 2 DIABETES IN BRAZIL: A MODELLING STUDY

Patrícia Vasconcelos Leitão Moreira[1*], Adélia da Costa Pereira de Arruda Neta[1], Flávia Emília Leite Lima Ferreira[1], Jevuks Matheus de Araújo[2], Rômulo Eufrosino de Alencar Rodrigues[2], Rafaela Lira Formiga Cavalcanti de Lima[1], Rodrigo Pinheiro de Toledo Vianna[1], José Moreira da Silva Neto[4], Martin O'Flaherty[5]

[1] Department of Nutrition, Federal University of Paraiba, João Pessoa, Paraíba, Brazil.
[2] Department of Economy, Federal University of Paraiba, João Pessoa, Paraíba, Brazil.
[3] Department of Nutrition, University of Sao Paulo, São Paulo, São Paulo, Brazil.
[4] Technical School of Health of the Federal University of Paraíba, João Pessoa, Paraíba, Brazil.
[5] Department of Public Health and Policy, University of Liverpool, Liverpool, United Kingdom.

## 1. The Model

The purpose of the “MODELA SUS” IMPACT Diabetes model is to provide estimates of future diabetes prevalence in Brazil and through different scenarios that have been proposed in relation to the obesity and smoking trend in Brazil, as well as the goals to face non-communicable diseases in the country, we provide a modelling basis for public policy makers.

MODELA SUS is a project that has been developed in Brazil in partnership with the National Council for Scientific and Technological Development (CNPq) for the development of a series of models related to diabetes, obesity, and cardiovascular diseases, in order to have scientific support for the establishment of public policies at the level of the Ministry of Health.

## Methods Overview

The model integrates information on population, obesity and smoking trends at a given point in time to estimate diabetes prevalence in the future. It was first developed for the EU Funded MedCHAMPS project, and applied in Tunisia, Turkey, Palestine, Syria and Saudi Arabia [1–5].

We adapted the model to the Brazilian context. Data on the Brazilian population were collected from the Brazilian Institute of Geography and Statistics (Instituto Brasileiro de Geografia e Estatística – IBGE), which provides data from the Population Census, as well as population projections until the year 2060.

Data on the prevalence of diabetes, obesity and smoking were collected from the study The Surveillance of Risk and Protective Factors for Chronic Diseases by Telephone Survey (VIGITEL) that makes up the Surveillance of Risk Factors for Chronic Non-Communicable Diseases (NCDs) system of the Ministry of Health.

In order to monitor the frequency and distribution of risk and protective factors for NCDs in all capitals of the 26 Brazilian states and in the Federal District since 2006. These diseases include diabetes, obesity, cancer, chronic respiratory and cardiovascular diseases such as arterial hypertension, which have a great impact on the population's quality of life [6].

The Brazilian population is partitioned in three states (healthy, obese and smokers) and from them, number of diabetes patients and diabetic and non-diabetic deaths are estimated for subsequent time periods using a Markov approach. The effect of policy decisions can be modelled by the estimated effect on risk factors trends, and the trend parameter can be modified to model decreasing or stable trends in the prevalence of obesity and or smoking.

## The Model Structure

We assume that the population can be divided in several pools: Diabetes mellitus, Obese, Smoker and “healthy” (eg: non obese, non-smokers, non-diabetics). A proportion of the population in each pool moves through pathways to other states as described in figure 1.

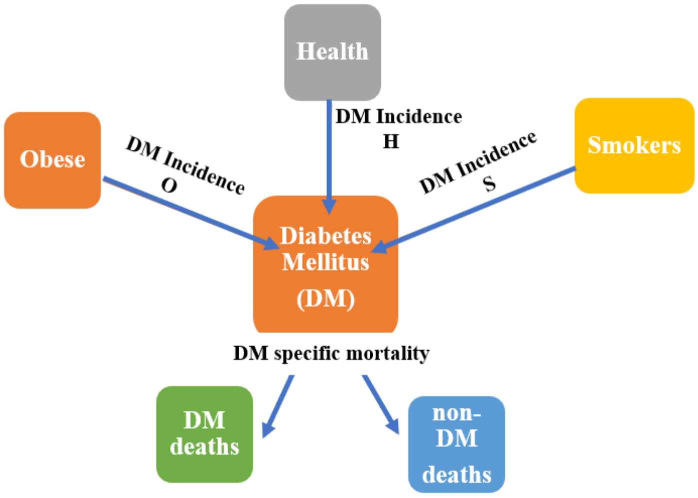


**Figure 1: The type 2 diabetes model structure**

## Model Validation

The validation exercise has been conducted, using the observed diabetes mellitus prevalence data between the years 2006 to 2020 with VIGITEL data in each of these years. As the VIGITEL data are self-reported, we adjusted a correction factor of 1.5 to prevent underestimations. Figures 2 and 3 shows the results for validation in men and women.

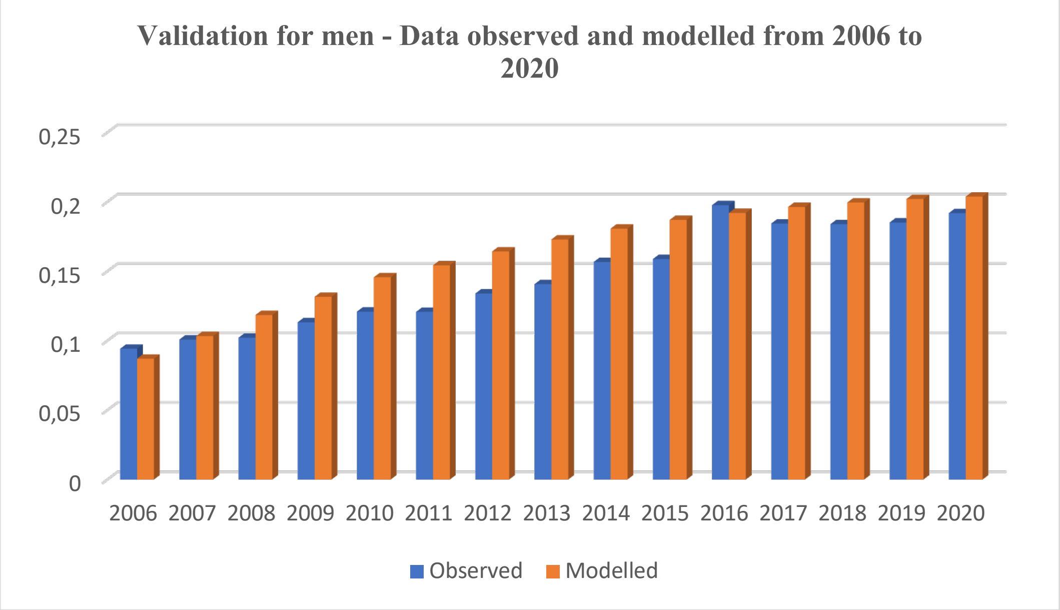


**Figure 2: Validation for men using data observed and modelled from 2006 to 2020.**

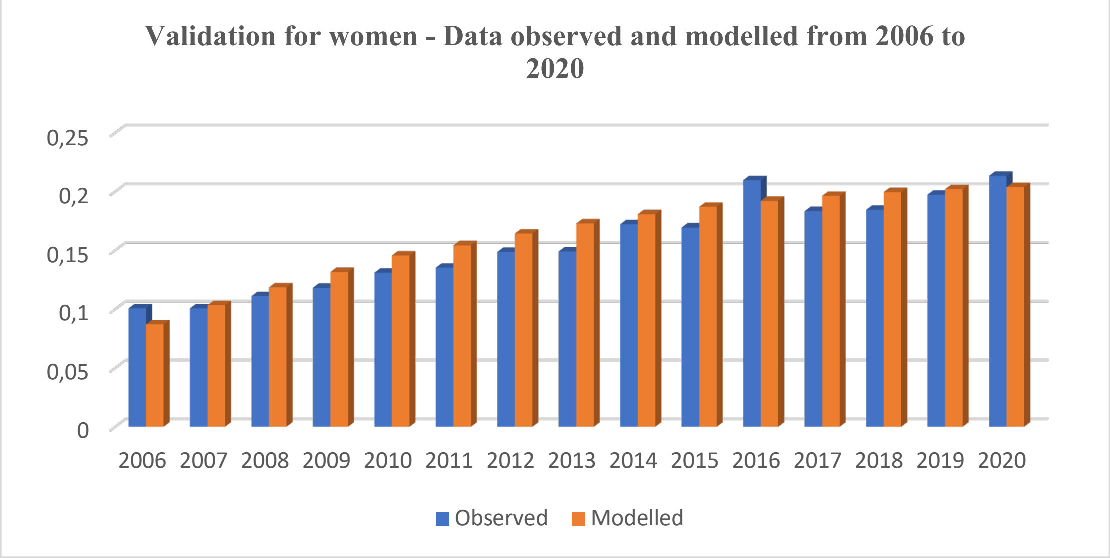


**Figure 3: Validation for women using data observed and modelled from 2006 to 2020.**

## DISMOD Inputs

In order to inform the model transition probabilities, we used DISMOD [7] to estimate incidence, case fatality and mortality using diabetes prevalence in 2010, assuming that the remission cases being equal to zero and the relative risks for mortality

We estimated the adjusted relative risk of mortality to input in DISMOD using the below formula,

$$RRadj = \frac{RR}{pRR + 1 - p} *$$

Where RR adj is the relative risk mortality, RR is the usual relative risk for mortality (mortality diseased/mortality healthy) and p is disease prevalence. The Verona Study [8] provides age and sex specific values for RR. A summary of the calculations for this parameter is presented in table1 and the formula used was:

* Based in the usual RR for mortality (mortality in diseased/mortality in non-diseased) and disease prevalence.

**Table 1: Estimating RR adj**

| Age and sex | Verona RR | DM Prevalence (2006) | RRadj |
|---|---|---|---|
| 25-34 | | | |
| men | 2,33 | 0,0135 | **2,29** |
| women | 3,43 | 0,0106 | **3,34** |
| 35-44 | | | |
| men | 2,33 | 0,0262 | **2,25** |
| women | 3,43 | 0,0239 | **3,24** |
| 45-54 | | | |
| men | 2,33 | 0,0703 | **2,13** |
| women | 3,43 | 0,0591 | **3,00** |
| 55-64 | | | |
| men | 2,13 | 0,1265 | **1,86** |
| women | 2,33 | 0,1350 | **1,98** |
| 65-74 | | | |
| men | 1,5 | 0,1691 | **1,38** |
| women | 2,27 | 0,1820 | **1,84** |
| 75+ | | | |
| men | 1,13 | 0,1833 | **1,10** |
| women | 1,32 | 0,1767 | **1,25** |

After DISMOD run, we obtained the DISMOD calculations presented in **Table 2** with results for Incidence, Case Fatality and Mortality. For DISMOD calculations we used the weights of: prevalence (50%), relative risk (100%) and remission zero. And the adjustments used for the inputs were quadratic and polynomial [9].

## Table 2: DISMOD run results.

| DISMOD RUN parameter set up: Trend 3% men, 3% women based on diabetes trends | | | | | | | | |
|---|---|---|---|---|---|---|---|---|
| Prevalence source: 2010 | | | | Weights: P 0.5, RR 1, R0 | | | | |
| Remission: 0 | | | | Trend: 10 year 3% men and women, assumed | | | | |
| RR mortality: specific for age | | | | Input adjustments: quadratic, polynomial | | | | |
| **Incidence** | | | **Case Fatality Rate*** | | | **Mortality** | | |
| **25-34** | 0,0300 | 0,0693 | **25-34** | 0,0004 | 0,0005 | **25-34** | 0,0001 | 0,0003 |
| **35-44** | 0,0273 | 0,0926 | **35-44** | 0,0013 | 0,0016 | **35-44** | 0,0006 | 0,0012 |
| **45-54** | 0,0202 | 0,0996 | **45-54** | 0,0038 | 0,0042 | **45-54** | 0,0021 | 0,0037 |
| **55-64** | 0,0101 | 0,1000 | **55-64** | 0,0111 | 0,0100 | **55-64** | 0,0064 | 0,0094 |
| **65-74** | 0,0038 | 0,1000 | **65-74** | 0,0245 | 0,0197 | **65-74** | 0,0134 | 0,0190 |
| **75+** | 0,0005 | 0,0698 | **75+** | 0,0287 | 0,0241 | **75+** | 0,0135 | 0,0236 |

## Table 3: BMI data inputs

**Obesity prevalence Trends(BMI >30)**
Source: Surveillance of Risk and Protection Factors for Chronic Diseases by Telephone Survey (VIGITEL)

| | Men | | | | | | | Women | | | | | | |
|---|---|---|---|---|---|---|---|---|---|---|---|---|---|---|
| | 5 | 3 | 12 | 11 | 3 | 0 | | 0 | 2 | 2 | 1 | 1 | 3 | |
| **year** | **25-34** | **35-44** | **45-54** | **55-64** | **65-74** | **75+** | **weighted avg** | **25-34** | **35-44** | **45-54** | **55-64** | **65-74** | **75+** | **weighted avg** |
| **2006** | 0,122 | 0,155 | 0,173 | 0,161 | 0,132 | 0,089 | 0,145 | 0,071 | 0,105 | 0,148 | 0,190 | 0,190 | 0,183 | 0,125 |
| **2007** | 0,131 | 0,172 | 0,196 | 0,183 | 0,136 | 0,086 | 0,159 | 0,075 | 0,116 | 0,171 | 0,195 | 0,187 | 0,172 | 0,133 |
| **2008** | 0,129 | 0,173 | 0,186 | 0,180 | 0,136 | 0,093 | 0,157 | 0,083 | 0,123 | 0,178 | 0,208 | 0,197 | 0,187 | 0,142 |
| **2009** | 0,133 | 0,169 | 0,186 | 0,188 | 0,154 | 0,124 | 0,161 | 0,093 | 0,141 | 0,175 | 0,208 | 0,202 | 0,166 | 0,149 |
| **2010** | 0,149 | 0,195 | 0,210 | 0,205 | 0,152 | 0,134 | 0,179 | 0,113 | 0,148 | 0,190 | 0,216 | 0,201 | 0,184 | 0,162 |
| **2011** | 0,162 | 0,217 | 0,217 | 0,197 | 0,159 | 0,100 | 0,188 | 0,116 | 0,163 | 0,205 | 0,225 | 0,205 | 0,181 | 0,171 |
| **2012** | 0,169 | 0,208 | 0,212 | 0,206 | 0,170 | 0,116 | 0,190 | 0,129 | 0,167 | 0,208 | 0,233 | 0,228 | 0,212 | 0,182 |
| **2013** | 0,178 | 0,228 | 0,229 | 0,213 | 0,179 | 0,134 | 0,203 | 0,126 | 0,170 | 0,198 | 0,218 | 0,220 | 0,198 | 0,176 |
| **2014** | 0,170 | 0,221 | 0,218 | 0,220 | 0,165 | 0,139 | 0,197 | 0,141 | 0,185 | 0,208 | 0,228 | 0,230 | 0,199 | 0,189 |
| **2015** | 0,178 | 0,243 | 0,216 | 0,216 | 0,166 | 0,128 | 0,204 | 0,141 | 0,188 | 0,209 | 0,224 | 0,229 | 0,213 | 0,190 |
| **2016** | 0,182 | 0,251 | 0,236 | 0,220 | 0,186 | 0,125 | 0,213 | 0,140 | 0,177 | 0,207 | 0,229 | 0,222 | 0,219 | 0,187 |
| **2017** | 0,173 | 0,245 | 0,243 | 0,223 | 0,194 | 0,143 | 0,212 | 0,132 | 0,189 | 0,200 | 0,219 | 0,218 | 0,203 | 0,184 |
| **2018** | 0,189 | 0,257 | 0,233 | 0,213 | 0,191 | 0,131 | 0,216 | 0,149 | 0,185 | 0,216 | 0,240 | 0,232 | 0,216 | 0,197 |
| **2019** | 0,197 | 0,242 | 0,247 | 0,235 | 0,196 | 0,146 | 0,221 | 0,163 | 0,204 | 0,221 | 0,234 | 0,227 | 0,212 | 0,205 |
| **2020** | 0,201 | 0,259 | 0,255 | 0,244 | 0,197 | 0,142 | 0,229 | 0,180 | 0,224 | 0,231 | 0,235 | 0,237 | 0,208 | 0,217 |
| **2021** | 0,206 | 0,258 | 0,259 | 0,249 | 0,209 | 0,164 | 0,234 | 0,160 | 0,203 | 0,235 | 0,257 | 0,252 | 0,234 | 0,215 |
| **2022** | 0,211 | 0,263 | 0,264 | 0,254 | 0,215 | 0,169 | 0,239 | 0,165 | 0,207 | 0,239 | 0,262 | 0,257 | 0,238 | 0,220 |
| **2023** | 0,217 | 0,268 | 0,270 | 0,259 | 0,220 | 0,174 | 0,244 | 0,170 | 0,212 | 0,244 | 0,266 | 0,261 | 0,243 | 0,225 |
| **2024** | 0,222 | 0,273 | 0,275 | 0,265 | 0,225 | 0,180 | 0,249 | 0,174 | 0,216 | 0,249 | 0,271 | 0,266 | 0,248 | 0,230 |
| **2025** | 0,227 | 0,279 | 0,280 | 0,270 | 0,230 | 0,185 | 0,255 | 0,179 | 0,221 | 0,253 | 0,275 | 0,270 | 0,252 | 0,235 |
| **2026** | 0,232 | 0,284 | 0,285 | 0,275 | 0,236 | 0,190 | 0,260 | 0,184 | 0,226 | 0,258 | 0,280 | 0,275 | 0,257 | 0,240 |
| **2027** | 0,237 | 0,289 | 0,290 | 0,280 | 0,241 | 0,195 | 0,265 | 0,188 | 0,230 | 0,262 | 0,285 | 0,280 | 0,261 | 0,245 |
| **2028** | 0,243 | 0,294 | 0,296 | 0,285 | 0,246 | 0,201 | 0,270 | 0,193 | 0,235 | 0,267 | 0,289 | 0,284 | 0,266 | 0,250 |
| **2029** | 0,248 | 0,300 | 0,301 | 0,291 | 0,251 | 0,206 | 0,275 | 0,197 | 0,240 | 0,272 | 0,294 | 0,289 | 0,271 | 0,255 |
| **2030** | 0,253 | 0,305 | 0,306 | 0,296 | 0,256 | 0,211 | 0,280 | 0,202 | 0,244 | 0,276 | 0,298 | 0,293 | 0,275 | 0,260 |
| **2031** | 0,258 | 0,310 | 0,311 | 0,301 | 0,262 | 0,216 | 0,285 | 0,207 | 0,249 | 0,281 | 0,303 | 0,298 | 0,280 | 0,265 |
| **2032** | 0,264 | 0,315 | 0,317 | 0,306 | 0,267 | 0,222 | 0,291 | 0,211 | 0,253 | 0,285 | 0,308 | 0,303 | 0,284 | 0,270 |
| **2033** | 0,269 | 0,320 | 0,322 | 0,312 | 0,272 | 0,227 | 0,296 | 0,216 | 0,258 | 0,290 | 0,312 | 0,307 | 0,289 | 0,275 |
| **2034** | 0,274 | 0,326 | 0,327 | 0,317 | 0,277 | 0,232 | 0,301 | 0,220 | 0,263 | 0,295 | 0,317 | 0,312 | 0,294 | 0,280 |
| **2035** | 0,279 | 0,331 | 0,332 | 0,322 | 0,283 | 0,237 | 0,306 | 0,225 | 0,267 | 0,299 | 0,321 | 0,316 | 0,298 | 0,285 |
| **2036** | 0,285 | 0,336 | 0,338 | 0,327 | 0,288 | 0,243 | 0,311 | 0,230 | 0,272 | 0,304 | 0,326 | 0,321 | 0,303 | 0,291 |

**Table 4: Transition probabilities matrix for each age in men (Markov Chain)**

| Transition probabilities matrix - men 25-34 years old | | | | | | |
|---|---|---|---|---|---|---|
| | **H*** | **O**** | **S***** | **DM♣** | **D, No DM#** | **D, DM##** |
| **H*** | 0,773515049 | 0,22 | 0,21 | 0,006384956 | 9,9995E-05 | |
| **O**** | | 0,9571208 | | 0,042779205 | 9,9995E-05 | |
| **S***** | | | | 0,009194337 | 9,9995E-05 | |
| **DM♣** | | | | 0,997802208 | 9,9995E-05 | 0,002097797 |
| **D, No DM#** | | | | | | |
| **D, DM##** | | | | | | |

| Transition probabilities matrix - men 35-44 years old | | | | | | |
|---|---|---|---|---|---|---|
| | **H*** | **O**** | **S***** | **DM♣** | **D, No DM#** | **D, DM##** |
| **H*** | 0,773515049 | 0,22 | 0,21 | 0,007444975 | 9,9995E-05 | |
| **O**** | | 0,9571208 | | 0,049881331 | 9,9995E-05 | |
| **S***** | | | | 0,010720764 | 9,9995E-05 | |
| **DM♣** | | | | 0,996205853 | 9,9995E-05 | 0,002097797 |
| **D, No DM#** | | | | | | |
| **D, DM##** | | | | | | |

| Transition probabilities matrix - men 45-54 years old | | | | | | |
|---|---|---|---|---|---|---|
| | **H*** | **O**** | **S***** | **DM♣** | **D, No DM#** | **D, DM##** |
| **H*** | 0,769122636 | 0,22 | 0,21 | 0,009878 | 0,001 | |
| **O**** | | 0,932819 | | 0,066182 | 0,001 | |
| **S***** | | | | 0,014224 | 0,001 | |
| **DM♣** | | | | 0,992919 | 0,001 | 0,006081 |
| **D, No DM#** | | | | | | |
| **D, DM##** | | | | | | |

| Transition probabilities matrix - men 55-64 years old | | | | | | |
|---|---|---|---|---|---|---|
| | **H*** | **O**** | **S***** | **DM♣** | **D, No DM#** | **D, DM##** |
| **H*** | 0,767601037 | 0,22 | 0,21 | 0,010301 | 0,002098 | |
| **O**** | | 0,928884 | | 0,069018 | 0,002098 | |
| **S***** | | | | 0,014834 | 0,002098 | |
| **DM♣** | | | | 0,987358 | 0,002098 | 0,010544 |

| | | | | | | |
|---|---|---|---|---|---|---|
| **D, No DM#** | | | | | | |
| **D, DM##** | | | | | | |

| **Transition probabilities matrix - men 65-74 years old** | | | | | | |
|---|---|---|---|---|---|---|
| | **H*** | **O**** | **S***** | **DM♣** | **D, No DM#** | **D, DM##** |
| **H*** | 0,7677383 | 0,22 | 0,21 | 0,009167 | 0,003095 | |
| **O**** | | 0,935489 | | 0,061416 | 0,003095 | |
| **S***** | | | | 0,0132 | 0,003095 | |
| **DM♣** | | | | 0,982904 | 0,003095 | 0,014001 |
| **D, No DM#** | | | | | | |
| **D, DM##** | | | | | | |

| **Transition probabilities matrix - men 75+ years old** | | | | | | |
|---|---|---|---|---|---|---|
| | **H*** | **O**** | **S***** | **DM♣** | **D, No DM#** | **D, DM##** |
| **H*** | 0,76655803 | 0,22 | 0,21 | 0,008255 | 0,005187 | |
| **O**** | | 0,939502 | | 0,055312 | 0,005187 | |
| **S***** | | | | 0,011888 | 0,005187 | |
| **DM♣** | | | | 0,972565 | 0,005187 | 0,022249 |
| **D, No DM#** | | | | | | |
| **D, DM##** | | | | | | |

*Health; **Obese; ***Smoking; ♣Diabetes mellitus; #Non-Diabetes related mortality; ##Diabetes related mortality.

**Table 5: Transition probabilities matrix for each age in women (Markov Chain)**

| **Transition probabilities matrix - women 25-34 years old** | | | | | | |
|---|---|---|---|---|---|---|
| | **H*** | **O**** | **S***** | **DM♣** | **D, No DM#** | **D, DM##** |
| **H*** | 0,778121555 | 0,22 | 0,21 | 0,001778 | 9,9995E-05 | |
| **O**** | | 0,977847 | | 0,022053 | 9,9995E-05 | |
| **S***** | | | | 0,002561 | 9,9995E-05 | |
| **DM♣** | | | | 0,998501 | 9,9995E-05 | 0,001399 |
| **D, No DM#** | | | | | | |
| **D, DM##** | | | | | | |

| **Transition probabilities matrix - women 35-44 years old** | | | | | | |
|---|---|---|---|---|---|---|
| | **H*** | **O**** | **S***** | **DM♣** | **D, No DM#** | **D, DM##** |
| **H*** | 0,777598718 | 0,22 | 0,21 | 0,002101 | 0,0003 | |
| **O**** | | 0,973644 | | 0,026056 | 0,0003 | |
| **S***** | | | | 0,003026 | 0,0003 | |

| | | | | | | |
|---|---|---|---|---|---|---|
| **DM♣** | | | | 0,996405 | 0,0003 | 0,003295 |
| **D, No DM#** | | | | | | |
| **D, DM##** | | | | | | |

| **Transition probabilities matrix - women 45-54 years old** | | | | | | |
|---|---|---|---|---|---|---|
| | **H*** | **O**** | **S***** | **DM♣** | **D, No DM#** | **D, DM##** |
| **H*** | 0,776818838 | 0,22 | 0,21 | 0,002282 | 0,0009 | |
| **O**** | | 0,970809 | | 0,028291 | 0,0009 | |
| **S***** | | | | 0,003285 | 0,0009 | |
| **DM♣** | | | | 0,99282 | 0,0009 | 0,00628 |
| **D, No DM#** | | | | | | |
| **D, DM##** | | | | | | |

| **Transition probabilities matrix - women 55-64 years old** | | | | | | |
|---|---|---|---|---|---|---|
| | **H*** | **O**** | **S***** | **DM♣** | **D, No DM#** | **D, DM##** |
| **H*** | 0,775643907 | 0,22 | 0,21 | 0,002757 | 0,001599 | |
| **O**** | | 0,96421 | | 0,034191 | 0,001599 | |
| **S***** | | | | 0,003971 | 0,001599 | |
| **DM♣** | | | | 0,990235 | 0,001599 | 0,008166 |
| **D, No DM#** | | | | | | |
| **D, DM##** | | | | | | |

| **Transition probabilities matrix - women 65-74 years old** | | | | | | |
|---|---|---|---|---|---|---|
| | **H*** | **O**** | **S***** | **DM♣** | **D, No DM#** | **D, DM##** |
| **H*** | 0,772302053 | 0,22 | 0,21 | 0,003407 | 0,004291 | |
| **O**** | | 0,95346 | | 0,042249 | 0,004291 | |
| **S***** | | | | 0,004906 | 0,004291 | |
| **DM♣** | | | | 0,977674 | 0,004291 | 0,018035 |
| **D, No DM#** | | | | | | |
| **D, DM##** | | | | | | |

| **Transition probabilities matrix - women 75+ years old** | | | | | | |
|---|---|---|---|---|---|---|
| | **H*** | **O**** | **S***** | **DM♣** | **D, No DM#** | **D, DM##** |
| **H*** | 0,760375257 | 0,22 | 0,21 | 0,007203 | 0,012422 | |
| **O**** | | 0,898266 | | 0,089312 | 0,012422 | |
| **S***** | | | | 0,010372 | 0,012422 | |
| **DM♣** | | | | 0,941284 | 0,012422 | 0,046294 |
| **D, No DM#** | | | | | | |
| **D, DM##** | | | | | | |

*Health; **Obese; ***Smoking; ♣Diabetes mellitus; #Non-Diabetes related mortality; ##Diabetes related mortality.

## References


1. Saidi O, O’Flaherty M, Mansour NB, Aissi W, Lassoued O, Capewell S, et al. Forecasting Tunisian type 2 diabetes prevalence to 2027: validation of a simple model. BMC Public Health. 2015;15:104.

2. Abu-Rmeileh NME, Husseini A, Capewell S, O’Flaherty M, on behalf of MEDCHAMPS project. Preventing type 2 diabetes among Palestinians: comparing five future policy scenarios. BMJ Open. 2013;3:e003558.

3. Awad SF, O’Flaherty M, El-Nahas KG, Al-Hamaq AO, Critchley JA, Abu-Raddad LJ. Preventing type 2 diabetes mellitus in Qatar by reducing obesity, smoking, and physical inactivity: mathematical modeling analyses. Popul Health Metr. 2019;17:20–20.

4. Al Ali R, Mzayek F, Rastam S, M Fouad F, O’Flaherty M, Capewell S, et al. Forecasting future prevalence of type 2 diabetes mellitus in Syria. BMC Public Health. 2013;13:507.

5. Sözmen K, Unal B, Capewell S, Critchley J, O’Flaherty M. Estimating diabetes prevalence in Turkey in 2025 with and without possible interventions to reduce obesity and smoking prevalence, using a modelling approach. International Journal of Public Health. 2015;60:13–21.

6. Ministry of Health. VIGITEL BRASIL 2020. Vigilância De Fatores De Risco E Proteção Para Doenças Crônicas Por Inquérito Telefônico. Brasília: Ministry of Health; 2021.

7. Barendregt JJ, Van Oortmarssen GJ, Vos T, Murray CJ. A generic model for the assessment of disease epidemiology: the computational basis of DisMod II. Popul Health Metr. 2003;1:4.

8. Muggeo M, Verlato G, Bonora E, Bressan F, Girotto S, Corbellini M, et al. The Verona diabetes study: a population-based survey on known diabetes mellitus prevalence and 5-year all-cause mortality. Diabetologia. 1995;38:318–25.

9. Barendregt JJ, Baan CA, Bonneux L. An Indirect Estimate of the Incidence of Non-Insulin-Dependent Diabetes Mellitus. Epidemiology. 2000;11.
